# Photothermal Optical Beam Steering Using Large Deformation Multi-Layer Thin Film Structures

**DOI:** 10.3390/mi12040428

**Published:** 2021-04-14

**Authors:** Harris J. Hall, Sean McDaniel, Piyush Shah, David Torres, Jose Figueroa, LaVern Starman

**Affiliations:** 1 Air Force Research Laboratory, Sensors Directorate, Wright-Patterson AFB, OH 45433, USA; sean.mcdaniel.1@us.af.mil (S.M.); david.torres-reyes.1@us.af.mil (D.T.); lavern.starman.1@us.af.mil (L.S.); 2 Apex Microdevices LLC, West Chester, OH 45431, USA; piyush.shah.1.ctr@us.af.mil; 3 KBR Wyle Corporation, Houston, TX 77002, USA; figuer57@msu.edu

**Keywords:** photothermal actuation, thin-film structures, micro-optics, optical beam control

## Abstract

Photothermal actuation of microstructures remains an active area of research for microsystems that demand electrically isolated, remote, on-chip manipulation. In this study, large-deformation structures constructed from thin films traditional to microsystems were explored through both simulation and experiment as a rudimentary means to both steer and shape an incident light beam through photothermal actuation. A series of unit step infrared laser exposures were applied at increasing power levels to both uniformly symmetric and deliberately asymmetric absorptive structures with the intent of characterizing the photothermal tilt response. The results indicate that a small angle (<4° at ~74 W/cm^2^) mechanical tilt can be instantiated through central placement of an infrared beam, although directional control appears highly sensitive to initial beam placement. Greater responsivity (up to ~9° mechanical tilt at ~54 W/cm^2^) and gross directional control was demonstrated with an asymmetrical absorptive design, although this response was accompanied by a large amount (~5–10°) of mechanical tilt burn-in and drift. Rigorous device cycling remains to be explored, but the results suggest that these structures, and those similar in construction, can be further matured to achieve controllable photoactuation suitable for optical beam control or other applications.

## 1. Introduction

Light-induced mechanical actuation has been of significant interest to the microsystems community for decades (see [1]) as it offers a remote, wireless means of power and control, which is necessary for autonomous design concepts that demand electrical isolation. At the micro- and mesoscales, applications cited in the literature include, but are certainly not limited to, drug delivery [2], microrobotic locomotion [3,4,5], haptic displays [6,7], scanning probe microscopy engines [8], actuation of tunable microlenses for endoscopy [9] and self-regulating mechanisms, such as automatic grippers [10], smart curtains/windows [11] and artificial self-adjusting optical iris [12]. Like other forms of stimuli-responsive actuation, which have been well summarized in recent review articles [13,14,15], the “smart” or “reconfigurable” device behavior that is being exploited is a result of both the material response and the structural design. At the microscale, the device fabrication is most commonly accomplished through conventional microfabrication methods based upon UV lithography being applied to layered thin films, although localized modification and additive manufacturing have also become more prevalent through direct laser writing [13].

For most devices operating in a non-aqueous environment, the photothermal actuation response is traditionally predicated upon light being absorbed near the surface of or within the film(s) generating heat which in turn modifies the internal stress profiles through the structure. This creation or relaxation of the stresses from the light absorption can impart structural deformation via thermal expansion effects from the device geometry alone, which enables designs to be made from a single material (such as polycrystalline silicon) [16,17,18]. However, this approach is somewhat limiting in terms of the amount of stress differential and subsequent deformation that can be produced within a given area. Thus, it is common for many designs in the literature to use dissimilar layered films and exploit the differences in the mechanical properties between them to further enhance actuation, namely the intrinsic stress and coefficient of thermal expansion [3,11,19]. While the overall device response varies depending upon the structure, the material response achieved through this traditional photothermal actuation is largely linear, as the stress and deformation scale proportional to temperature, making it useful for many applications that demand analog control.

In the last few decades, several groups have explored using carbon materials (i.e., carbon nanotubes, carbon nanoparticles, graphene oxide flakes) and various polymer blends to enhance the photoabsorption and photomechanical properties of the films used as a means to improve upon the overall photothermomechanical response [2,13,20,21,22,23]. Similarly, numerous research groups have been advancing materials that exhibit nonlinear photothermal responses to further expand capability; for instance, by triggering dynamics that exceed the limitations of thermal time constants or adding hysteresis to enable memory effects. These include shape memory alloys (e.g., nickel titanium) [19], shape memory polymers [5], liquid crystal polymers [12,24,25] and other phase change materials (e.g., vanadium oxide, paraffin) [5,21,26], some of which incorporate photo-chemical effects. Some of these novel material solutions have yet to be integrated with wafer-scale microfabrication techniques, making this a rich area for future device development.

In this study, we successfully demonstrated photothermal actuation of large out-of-plane multilayer structures that are capable of large-angle optical beam steering and shaping via control of a central, reflective, thin, Au-coated plate. The devices were fabricated using conventional surface micromachining films but employed a non-traditional structural geometry to achieve the desired photothermal response. The structural design employed was similar to the inverted series-connected devices reported by the Xie group [9,27,28,29,30,31] in that it involved a chain of series connected with out-of-plane S-shaped beams. However, our design incorporated stressed films on the surface of a structural layer, allowing them to be fabricated predominantly via a commercial foundry process. These structures were originally intended as electrostatically actuated prototype undercarriages for high fill-factor micromirror arrays capable of large angle 3DOF (tip–tilt–piston) motion [32,33]. We recently reported that these structures, unmodified, can be photothermally actuated through small AC modulation of an infrared illuminator, making it possible to measure the frequency response of the structure up to 1 kHz [34]. In this paper, we report the steady-state unit step behavior (low sampling rate) of these devices under broad Gaussian illumination and show that with the addition of a single patterned absorber coating, large-angle beam steering can be achieved towards a preferential direction autonomously. In addition, we show that heating effects associated with the central mirror cause its curvature to relax as the beam steering increases, resulting in simultaneous change of the reflected beam profile.

To our knowledge, photothermal actuation for the purpose of large-angle optical beam steering has not been reported before, nor any photothermal characterization, for these types of large-deformation structures. The demonstration we show of photothermal one-directional tilt actuation in this work, seems possible to achieve with a simpler highly stressed bilayer cantilever beam based approach with patterned optical coatings. However, our structure offers (1) mechanically reduced coupling of the actuator and the optical reflector, allowing each to be independently altered; (2) a more compressed area footprint by virtue of the successive interconnected S-beams; and (3) the potential for multi-directional (both tip and tilt) photothermal actuation with more sophisticated coating patterns being applied to the structure’s actuator arms. These qualities make it interesting for photothermal actuation design and characterization.

## 2. Design and Fabrication

**Figure 1:**
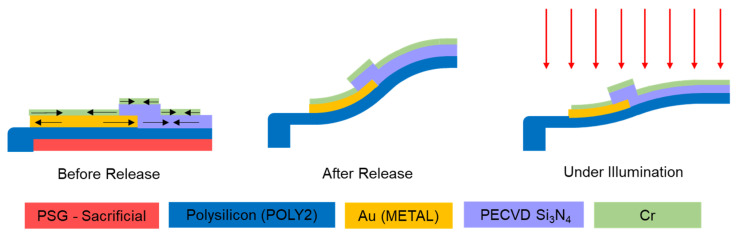
Simplified cross-section of a single multi-layer film cantilever with Cr coating.

The thin-film microstructures examined in this work were designed to achieve large out-of-plane deformation, upon removal of the sacrificial material (phosophosilicate glass), by leveraging the differences in constituent film stresses present after their deposition. These stresses are primarily an artifact of each layer’s mismatch in thermal expansion with respect to the underlying layer. The structures each spanned a ~1 mm^2^ area and were fabricated through the use of the PolyMUMPs^TM^ foundry process (specifically, the 1.5 µm thick POLY2 layer and 500 nm thick Au layer—see [35]) in conjunction with post-processing of additional lithographically patterned films. The first post-processed layer was 1-µm-thick non-stoichiometric Si_3_N_4_, deposited via plasma enhanced chemical vapor deposition (PECVD) (temperature = 300 °C, power = 60 W, pressure = 800 mT, SiHe = 167 sccm, N_2_ = 200 sccm, NH_3_ = 2 sccm, He = 600 sccm), which provided a compressively stressed structural layer that was selectively patterned. The Si_3_N_4_ patterning was accomplished by selectively masking the regions where material was desired with photoresist (AZ 4620) and then performing a reactive ion etch process (CF4 40 sccm, O2 3 sccm, 100 W, 50 mT). This etch had to be performed long enough to accommodate for any film non-uniformity and ensure removal of all the unwanted nitride. The second post-processed layer was a 200-nm-thick layer of evaporated chromium intended as a moderate broadband infrared absorber that was applied selectively to the structure via a conventional liftoff method, using a two-layer photoresist stack (LOR 10A liftoff layer and SPR 955.07 photoresist). All photoresists were deposited using conventional spin coating (4000 rpm, 30 ramp, 30 s) on each chip sample individually. Figure 1 provides an illustrative graphic for a single notional s-shaped cantilever using these films. The black arrows in Figure 1 indicate the primary film stress direction before release, assuming residual thermal film stress dominates based upon the coefficient of thermal expansion (CTE) values (see Table 1) for each film. The film stresses are more relaxed by the resultant S-shaped structural deformation after release. When illuminated by the infrared laser, the photothermal heating from the absorber layer and subsequent differential expansion of the films induces stresses in the reverse direction, allowing the deformation to flatten and overall downward motion at the end of the cantilever to occur.

**Figure 2:**
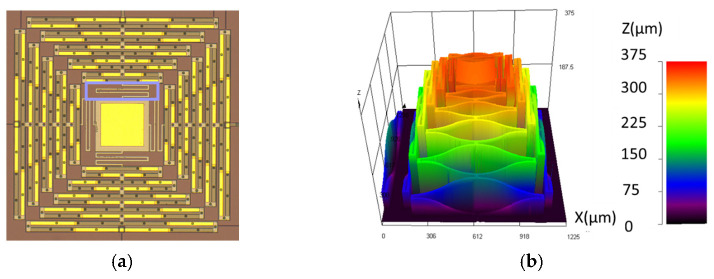

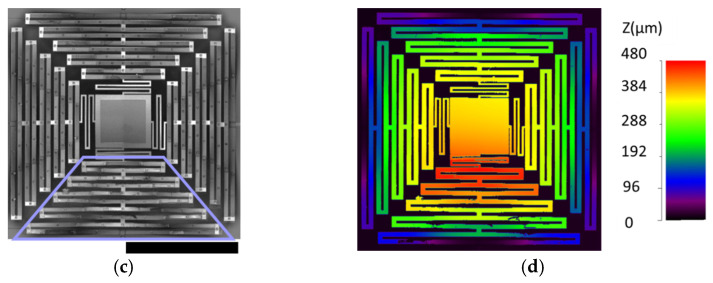
Selected top-view imagery of fabricated large-deformation, multi-layer, thin-film microstructures: (**a**) optical microscope image (×5) of uncoated (no Cr—device A) structure before release. A rectangular outline highlights one of the interconnect springs; (**b**) stitched 3D confocal microscope (3DCM) z-profile oblique view image (×20) of uncoated structure after release with z-deformation enhanced ×2.5 to accentuate visibility of the central plate curvature; (**c**) stitched multi-focus intensity image of structure with bottom arm Cr-coated (device C) after release. A trapezoidal outline highlights the Cr-coated arm. Inset black scale bar is 500 µm in length; (**d**) stitched 3DCM z-profile of structure with bottom arm Cr-coated after release.

The designs examined in this study were more intricate than a single cantilever, but predicated upon the same concept. A top view of the unreleased structure without the Cr coating is shown in Figure 2a. Each of the four serpentine polysilicon support arms were alternatively coated in Au (tensile stress) and silicon nitride (compressive stress) to create a meandering large-deformation profile in the z-axis as shown in Figure 2b. The arms were each connected to a simple intermediate spring structure made of uncoated polysilicon which in turn was connected to a square Au-coated polysilicon central plate, which served as a broadband optical reflector. The intermediate springs were intended to help decouple motion between the arms. In order to create an asymmetric photothermal response in the presence of a centrally positioned infrared laser, which is central for these structures to serve as large-angle beam-steering devices, the Cr layer was patterned uniformly onto the surface of a single arm. Figure 2c shows a 3D microscope intensity image constructed from a z-axis scan of a released structure, with the Cr patterned on the lower arm. The representative corresponding z-axis deformation profile is shown in Figure 2d.

**Figure 3:**
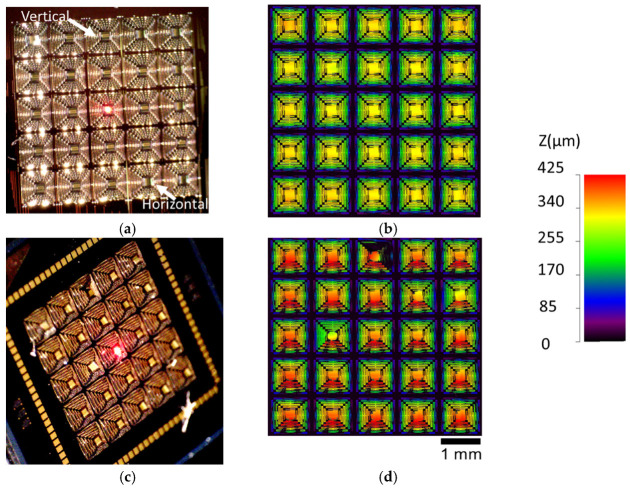
Fabricated 5 × 5 arrays of structures: (**a**) uncoated (no Cr), optical top view. All the elements had cylindrically curved central plates. The inset arrows point to example elements with curvature about the horizontal and the vertical axes of the plate, respectively; (**b**) uncoated (no Cr), height profile with color scale on right; (**c**) lower arm Cr-coated, optical oblique view; (**d**) lower arm Cr-coated, height profile with color scale on right.

**Figure 4:**
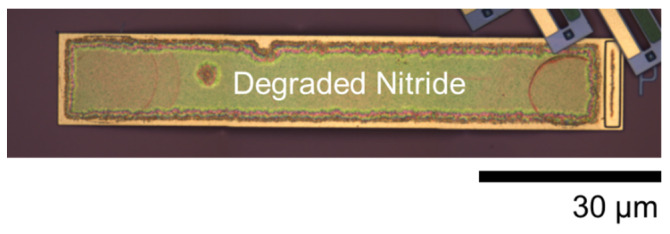
Optical microscope image of a polysilicon cantilever coated with Au and the post-processed silicon nitride layer after release in hydrofluoric acid (49%) Degradation of the nitride is evident.

**Figure 5:**
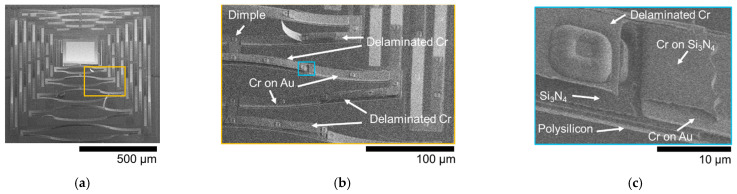
Scanning electron microscope images of a released device with the bottom arm coated with Cr; (**a**) 20° oblique device profile; (**b**) magnified image of Cr-coated arm (rectangular area in (a)) showing Cr delaminating from nitride; (**c**) magnified portion of a nitride on Au overlap region. The square imprinted feature is from a dimple patterned into the POLY2 layer. The Cr layer is clearly delaminated from the underlying nitride and the appearance of the nitride suggests chemical etching.

Two 5 × 5 arrays of devices were fabricated, the first with a single-arm Cr coating and the second without. In this work, we report the photothermal response for a few elements within each of these arrays to capture representative performance (both simulated and experimental). While the fabrication yield and uniformity were strong, and the structures were suitable for demonstrating the photothermal optical beam control intended, they did exhibit certain imperfections and features that are important to highlight to both provide proper context for the results and inform future related work. The first and arguably the most critical aspect to note is that the initial static deformation on the single-arm Cr-coated structures shown in Figure 2d and Figure 3d was not coincident with qualitative expectations. Ideally, the addition of the Cr film should both stiffen the arm that is coated and add film stresses that are counter to out-of-plane deformation, as indicated in Figure 1. The Cr-coated arm’s resultant static out-of-plane deformation should be less than that of the other arms, resulting in a downward tilt of the plate toward the coated arm. The fabricated results shown indicate that the opposite occurred, with the coated arm being effectively less stiff. Scanning electron microscope imagery and evidence from additional test structures suggest two likely causes, which are likely coupled. These are the galvanic corrosion of the nitride layer during release (49% hydrofluoric acid (HF) immersion) and partial delamination of the Cr layer in some locations. Galvanic corrosion of polysilicon is a well-known issue in PolyMUMPs, and was effectively managed in the design of the metal and polysilicon layers for this work [35]. However, degradation of the silicon nitride was not anticipated and seemed to be evident to some degree whenever the nitride is in direct contact with a metal layer (Au or Cr). Figure 4 shows an image of a post-HF release on-chip POLY2 cantilever coated with both Au followed by silicon nitride. The image shows clear degradation (etching) of the nitride. The regions of nitride on the surface of the Au were very small and thus this effect was largely inconsequential for the uncoated arms. However, the Cr-coated arms seemed to show evidence of this degradation as well. Partial delamination of the Cr film, shown in Figure 5, occurred exclusively on portions of the structure where the Cr was deposited on the surface of the nitride. This observation further suggests reduced adhesion due to nitride corrosion, enabling the tensile film stress inherent to the Cr film deposition to cause delamination. While both these effects are clearly undesirable, they were not significant enough to prevent comparative demonstration of photothermal beam control. They are clearly important to consider, however, for future designs, and necessitated a need to determine appropriate effective material parameters for the implementation of the representative models from which comparative simulated performance was generated, as described in the following section.

**Figure 6:**
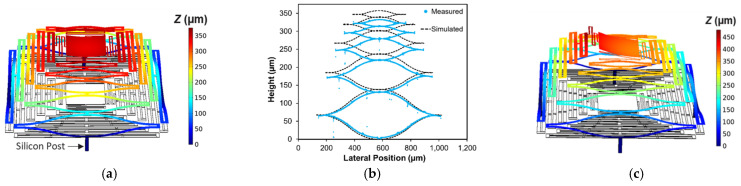
Simulated results of initial deformation profiles. (**a**) Baseline design; (**b**) comparison of height profiles for a single actuator of the baseline design vs. measured data shown in Figure 2b (device C); (**c**) Cr-coated single-arm design showing 12.8° initial central plate tilt (rotated 90° relative to Figure 2d to show tilt).

Another important feature to note for these structures (see Figure 5a and Figure 6) is that the central square polysilicon plate (250 µm width) with Au reflective coating (200 µm width, positioned on center), supported by the four serpentine arms, was not flat, but rather curved cylindrically (radius of curvature 700–775 µm). This cylindrical curvature is coincident with layered plate theory, specifically the nonlinear, non-symmetric elliptical deformation regime which results from the misfit strains between the films (film stress differences). Similar to the actuator arms, these stresses largely originate from the elevated deposition temperatures and the difference in thermal expansion between the films. An excellent explanation of this phenomena and relevant experimental results for near free-standing (centrally supported) gold-polysilicon square plates fabricated using the same PolyMUMPs process that is leveraged in this work is presented by Dunn et al. [36]. This elliptical deformation involves the triggering of a highly nonlinear bifurcation response, the onset and direction of which is influenced heavily by subtle perturbations that can include variations in the geometry or heterogeneities in the materials parameters. The gold-polysilicon square plates in [36] showed triggering of this response occurring between a larger width of 250 µm and 300 µm. The plates in this work differed in that they were not completely free standing, but connected to each of the four support arms by interconnect springs midway along each edge (highlighted in Figure 2a). These additional boundary conditions were a contributing factor for this difference in onset. Likewise, variations in the arms of these structures, which drive differences in the arm deformation, in turn acted as a major perturbation source which influenced the direction of bifurcation. As can be seen in Figure 3a, the direction of plate bifurcation across the 5 × 5 array varied between horizontal and vertical orientations. In this study we did not seek to deliberately manage the direction or magnitude of the plate curvature, but this would be a necessary consideration for batch fabrication and is feasible through asymmetric design of the interconnecting springs and the plate itself.

Cylindrical optical reflectors can be particularly advantageous for applications like 1D-LIDAR that seek to convert a Gaussian laser beam spot into a broader line spot (for a recent example, see [37]). The thin film-layered plate for the structure in this work would not likely be well suited for this application as it was very thin (~2 μm thick) relative to its size, making the curvature sensitive to temperature changes. Instead, this temperature sensitivity was a feature we exploited to show the simultaneous photothermal, optical beam-shaping capability in addition to large-angle beam steering, with the suggestion that each of these can be designed in a largely decoupled fashion.

Lastly, the subtractive patterning of the silicon nitride layer employed an etch chemistry that was not selective with respect to polysilicon, so the uncoated polysilicon regions of the structure, namely the interconnect springs, were also susceptible to etching once the nitride was removed. Contact profilometry performed indicated that these regions were etched at approximately 600 nm (~40% thickness reduction), reducing the effective stiffness of these springs. The simulations detailed in the following section incorporated this effect, although it was determined that the impact on steady-state piston and tilt displacements was minor. Nevertheless, this non-uniformity in etching is another source of perturbation variation for the aforementioned plate bifurcation and would impact the dynamic response (not examined in this work).

## 3. Modeling and Simulation

Creating models sufficient for predictive simulation of future designs of these structures, tailored for specific photothermomechanical responses, is important for enabling their use in devices. In this work, commercial finite element analysis-based multiphysics software (COMSOL v 5.4) was used to perform simulations of the initial unilluminated static deformation and the steady-state photothermal response of both the baseline design (no Cr coating) and single-arm Cr-coated designs.

### 3.1. Unilluminated Static Deformation

Generation of the initial static deformation model considered two primary factors, both in comparison to the measured deformation profiles shown in Figure 2—namely, the S-shape of the deformation profile of the actuators and the absolute height of the structure. The model used in this work employed a direct application of the effective biaxial film stresses to the layers, as opposed to an indirect generation of thermal stresses based on the deposition temperatures of each film. This approach allowed for independent assignment of the film stresses and subsequent parametric study in order to validate the model with the measured data. It is important to note that these simulation studies were limited to linear simulations and did not accommodate geometric nonlinearity effects in order to keep computational expense manageable. Thus, they did not incorporate the aforementioned nonlinear cylindrical bifurcation of the thin central plate. Figure 6a shows an oblique profile of the simulated initial deformation results for the baseline design and Figure 6b provides a comparison of simulation results for the single arm with the measured height profile shown in Figure 2b. These results indicated reasonable qualitative agreement for the actuator shape and were within a < ~8% deviation in absolute height (357 µm simulated, 332 µm measured), suggesting that the model is reasonable for capturing deformation. The mechanical materials constants and stress values used to generate these results are shown in Table 1.

It is important to note that the values for the Young’s modulus and film stress shown in Table 1 are very much “effective” quantities and are not necessarily representative of the actual material parameters in many instances. For example, the silicon nitride was measured to be compressive in nature through wafer bow measurements (~−600–−800 MPa), which is not in agreement with the −1 GPa used in the simulation. Likewise, while the Au layer is representative of values reported for the PolyMUMPs process, the polysilicon stress, which has consistently been reported as being ~10 MPa, is clearly not [35,38]. In similar fashion, the single-arm Cr-coated structural model, shown in Figure 6c, utilized a separate “effective” nitride layer with a much lower Young’s modulus to capture the galvanic corrosion effects. In addition, this model used a single Cr layer with a common biaxial stress value, when a more appropriate representation would have different stress values for the portions of Cr that cover the nitride and Au, respectively, as indicated by Figure 2a and the CTE values in Table 1, which would dictate different thermal stresses upon deposition. Despite these significant deviations in material parameters, the models were nevertheless effective at creating reasonable quantitative agreement with the measured results. It is obvious, however, that there is significant opportunity to improve upon them for ab initio prediction.

### 3.2. Photothermal Steady-State Deformation Response

Modification of the initial static deformation model with the appropriate thermal physics allowed for coupled thermo-mechanical simulations of the steady-state photothermal actuation of the structure. Laser heating was incorporated purely through the use of surface heat sources (incident heat flux) proportional to that expected from a centrally positioned Gaussian beam (450 µm radius).

For the portions of the structure with a metal surface (Au or Cr), this was an accurate approach as the penetration depths of the infrared light were very shallow (<100 nm). For the portions of the structure that were semi-transparent, the total absorption through the thin layer stack was estimated using a simple application of Beer’s Law to create an effective emissivity for the surface. This approach is advantageous in its simplicity, as the power absorbed is a fraction of the incident power independent of wavelength or angle of incidence. The subsequent model response can thus be tuned rather easily through parametric adjustment of the emissivity (absorption) values used. However, it is critical to recognize that, for semi-transparent stacks, it fails to capture full wave effects, such as interference and Fabry–Perot resonance, which can dramatically affect the absorption.

In this study, as described in the following section, the laser wavelength used to impart photothermal heating was 1532 nm. At this wavelength the Au is known to be highly reflective [39] and the Cr is also recognized as a moderate absorber [40]. Studies from the literature suggest that the 1-µm-thick silicon nitride layer is entirely transparent [41] and that the POLY2 layer, based upon its doping density [42], has an absorption coefficient of 400 cm^−1^ [43], which equates to a total absorption of ~5.8% for a 1.5-µm-thick layer. This value was used as the surface emissivity for the POLY2/Si_3_N_4_ regions of the baseline actuator. Optical simulations of this film stack in our prior work [34] indicated the potential for absorption beyond this value, although experimental verification of this was not feasible. The emissivity (absorption) of the Cr layer at 1532 nm for normal incidence was determined through infrared spectroscopy (not shown) to be approximately 0.30, which is in reasonable agreement with values suggested in the literature [44,45]. Higher values (0.35 and 0.4) were also examined to establish expectations for higher effective absorption due to possible variations in film quality and to facilitate subsequent comparison to experimental results.

**Figure 7:**
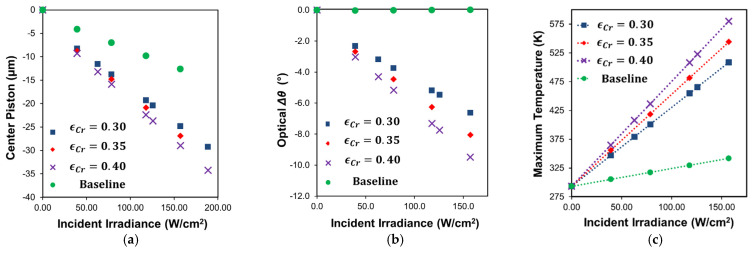
Simulated results of steady-state photothermal actuation of the baseline and single-arm Cr-coated structure at different effective Cr emissivity values. (**a**) Piston response (change in z-displacement) measured at center of plate. The unilluminated center plate height position is 335.4 μm for the baseline and 372.8 μm for the single-arm Cr-coated structure; (**b**) 1D tilt response of plate (change in single axis tilt from initial position). The unilluminated center plate initial mechanical tilt is 0° for the baseline and 12.8° for the single-arm Cr-coated structure; (**c**) maximum temperatures at each condition.

Conductive heat losses through the substrate were accommodated for with solid silicon posts (50 µm × 50 µm × 100 µm) added to the central anchor points of each actuation arm (visible in Figure 6a,c), to which fixed (room temperature) boundary conditions were applied to the backside. Additional heat conduction (800 W/m^2^∙K) through the air to the substrate from all the surfaces underneath the structure was included as a secondary loss mechanism, although the impact was minor. The relevant material thermal properties for all film layers are summarized in Table 1. Figure 7a,b present both the baseline and single-arm Cr-coated simulated piston (z-displacement towards the substrate) and tilt responses, respectively, for parametric sweeps of incident laser power at the different Cr emissivity values. Both structures clearly exhibit downward piston motion as the structure collapses from the reduction in the thermal stress mismatch in the film stack. However, as intended, the single-arm Cr-coated structure exhibits increasing change in 1D tilt (in the direction of the coated arm) as it collapses due to the asymmetry in optical absorption between the Cr-coated arm and the remainder of the structure. The baseline structure tilt response remains unchanged as irradiance increases. It is important to recognize that tilt motion is coupled to the piston motion for these devices, as the tilt is achieved through preferential collapse of one side of the structure, which causes both downward and lateral motion of the mirror plate in addition to tip/tilt rotation. For the single-arm coated structure, the magnitude of piston and tilt changes is comparatively larger due to the greater degree of overall absorption and subsequently higher steady-state temperatures throughout, as evidenced by their higher maximum temperatures shown in Figure 7c. While not presented, it is worth recognizing that asymmetric tilt was also clearly possible in simulation of the baseline structures (those without Cr coating) by directly steering the incident radiation (adding offset to the center Gaussian beam position) towards the desired steering direction.

## 4. Experimental Setup and Methods

The photothermally actuated unit step mechanical response of these structures under direct near-infrared (λ = 1532 nm) illumination was captured experimentally with a low sampling rate (~13.1 Hz) to quantify the steady-state behavior. As will be explained, additional video data processing of recorded visible laser spot behavior was necessary to extract the results.

### 4.1. Laser Heating Setup

**Figure 8:**
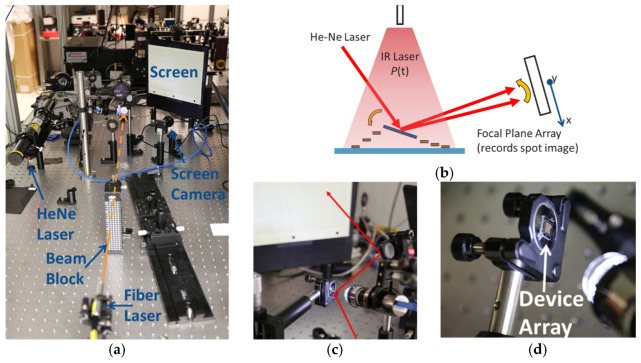
Experimental setup. (**a**) Overall optical test layout with the infrared laser beam path indicated by the orange line; (**b**) simple graphical depiction of photothermal actuation of the structure; (**c**) test image showing qualitative HeNe laser beam path with surrogate relay mirror (actual mirror much larger to accommodate large angular motion); (**d**) close-up image of device array under illumination of microscope camera.

The experimental setup used for these measurements is illustrated in Figure 8a–d. Essentially, an infrared fiber laser (continuous wave, IPG Photonics, Oxford, MA, USA) was positioned to be perpendicularly incident to illuminate the entire structure under test (450 µm Gaussian beam radius). This laser provided the optical energy for the photothermal heating of the structure and was controlled by a manual beam block (shutter). A second visible HeNe laser (λ = 632 nm, 100 µm Gaussian beam radius) was positioned at a small off-perpendicular angle (~8°) and focused on the central reflective platform of the structure. The Gaussian spot profiles of both lasers were each measured using beam profilers at the plane of the structure under test to accommodate for any impact on beam shape from the preceding relay optics. The exact focusing and beam divergence of the beams was not measured, but the beam radius error was estimated to be within +/− 2 μm based upon profiler measurements made and the precision of the z-stage used to mount the sample. The reflection of the HeNe laser off the platform was then relayed to another large, flat mirror in order to project the resulting spot onto a screen with a 1 cm spaced grid. Ideally, the visible laser would be perpendicular to the sample to completely eliminate the possibility of piston motion impacting the beam spot position on screen, but at maximum piston (i.e., complete collapse of the structure) the small offset angle only allowed for <70 μm of on-screen position error, making this effect inconsequential. Minor adjustments to the screen position were made for different elements to accommodate capturing the spot position, and the total optical path length of the HeNe beam was recorded at the start of each measurement. The path length was used to properly scale the recorded on-screen imaging in terms of tilt angle. A digital camera (Thor Labs, full frame rate: 13.1 frames/s) was positioned to allow for video recording of the reflected HeNe spot profile and spot position on the screen. The ideal position for this camera would be perpendicular to the screen to prevent any image distortion effects; however, the required optics in the setup necessitated an off-normal viewing (up to 52°) and thus additional video processing was performed to compensate, which is discussed in Section 4.2. As expected, the initial reflected spot profile on the screen was linear because of the curvature associated with the central plate. A second microscope camera (Dino-Lite, AnMo Electronics Corporation, Hsinchu, Taiwan, ROC) was positioned above the structure under test in order to record observed motion directly and observe any onset of damage.

**Figure 9:**
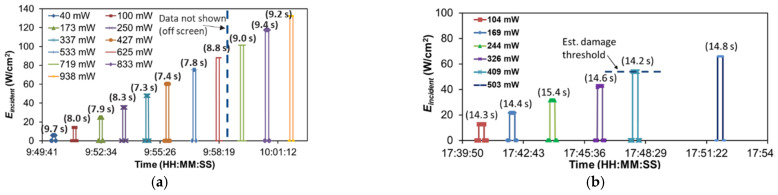
Actual test flows executed with exposure times shown in parenthesis above each exposure. (**a**) Baseline structure (no Cr coating—device A) with ~7–10 s exposure times; (**b**) single-arm Cr-coated structure (device C) with ~14–15 s exposure times. Each exposure ties to an individual test data collection point.

Experimental conduct for a given test point involved establishing a target laser power setting, measuring the laser power with an optical power meter, starting the screen camera and microscope camera recording manually (near simultaneously) and, after a few hundred frames, sharply removing the beam block to create optical unit step input. After several hundreds of frames the beam block was sharply returned to its initial position and a few hundred more frames were recorded to capture burn-in offsets in both position and shape before the recordings were stopped. The flow of testing for a given structure generally began with low infrared laser power and was increased to higher powers incrementally in a build-up manner sometimes resulting in destruction of the structure. As the beam block was manually placed, there was some variation in exposure times, but they were kept qualitatively consistent throughout a given test run. Date and time stamps for the recorded files coupled with analysis of the video data allowed for accurate mapping of the test flow and calculation of exact exposure times at each power level. The test flows performed on the uncoated structure shown in Figure 2b (device A) and the single-arm Cr-coated structure in Figure 2d (device C) are shown in Figure 9a,b as representative examples. Figure S1a,b (in the Supplementary Materials) show the remaining devices B and D test flows. Overall, the baseline structures were exposed for a shorter time duration (7–10 s) versus the single-arm Cr-coated structures (14–15 s). Table 2 presents a short summary of the devices tested with these differences. Since the aim of this experimentation was to measure steady-state motion and the corresponding temperature rise times were all << 1 s, this difference was not considered critical. However, with the presence of slow thermal drift, it is expected that the additional exposure times would drive slightly higher peak temperatures during each exposure.

### 4.2. Data Analysis

**Figure 10:**
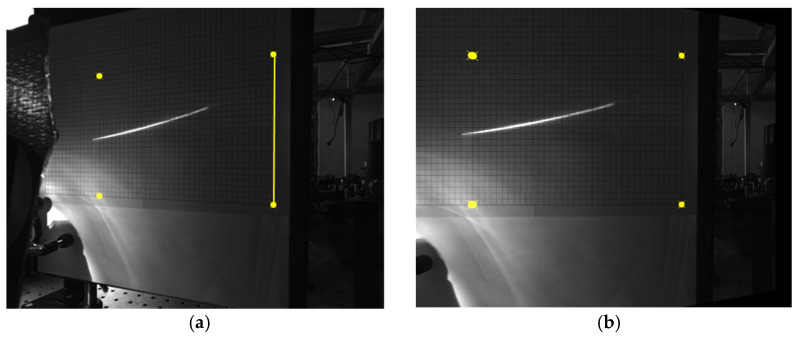
Example of full-frame perspective rectification for off-normal (~52°) camera viewing. (**a**) Raw data with distorted reference points used to create H matrix indicated. The yellow vertical line serves as a fixed distance reference to scale the transformation; (**b**) after warping with same reference points in desired normal perspective. Both images shown have had brightness enhanced by 40% to improve visibility.

The recorded video from the camera viewing the screen was processed on an individual frame-by-frame basis using a combination of scripted processing in ImageJ (version 1.52) and MATLAB (version 2020a). There were three main processing tasks performed on all the video datasets collected. The first was to correct for the off-axis viewing angle of the camera. This task was accomplished by creating a planar homography matrix using reference points inherent to the rectangular grid on the paper. The raw image was then warped by applying this matrix, effectively “moving” the camera so it was perpendicular to the screen. The processing was performed using the MATLAB Machine Vision Toolbox created by Corke and additional detail on it can be found in his textbook [46]. No additional information was added to the image, so areas more distant from the camera were more sparsely sampled in space, but the effects of this were minimal for the purposes of this analysis. Figure 10 provides a representative example of the perspective rectification performed for a single frame.

**Figure 11:**
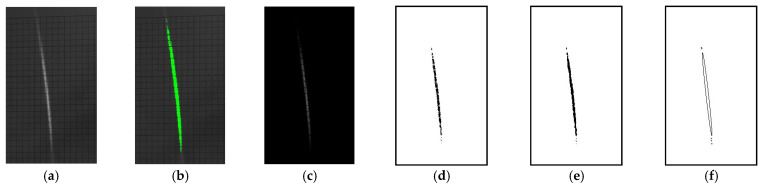
Frame image processing example. (**a**) Cropped raw data (**b**) with pixels above threshold highlighted in green; (**c**) after threshold and background subtraction; (**d**) converted to binary data; (**e**) after binary close operation; (**f**) after particle analysis (ellipsoid fitting). Note: this example utilized a near-normal (>80°) viewing angle but was not perspective-rectified and was cropped much smaller from the actual size to ease visualization.

Once the image perspective was adjusted for a given frame, the two remaining processing tasks, calculation of the laser spot position and the capturing of the shape of the spot (ellipsoid fitting), could be performed. The laser spot position was determined by first cropping raw data to an area that was sufficient for capturing the full range of motion of the spot and then performing background subtraction and image thresholding to remove image content unrelated to the spot (see Figure 11a–c). Some datasets required prior, additional, tailored dark-box overlaying to further ensure a dark (zero pixel value) background. The center of mass (CoM) of the resulting 8-bit grayscale weighted frame was then calculated using standard image measurement functionality inherent to ImageJ [47]. The CoM values were averaged across time periods before, during and after laser illumination for calculation of relative and absolute motion parameters, as well as any burn-in motion (plastic tip/tilt deformation) from the exposure. These pixel coordinates were converted into reflected vertical tip/horizontal tilt optical angles by applying simple trigonometry based upon the screen distance. The angular motion reported in this work uses a polar format display of the spot motion. The absolute magnitude of this tip/tilt motion, which we label as “Optical Tilt Angle Magnitude”, is the radial distance, and the polar angle is the screen in-plane angular position in relation to the origin. Mapping the spot position in this manner is useful to portray cross-coupled motion between tip and tilt.

Lastly, the spot shape was considered for each dataset as a quantitative measure of how the curvature of the platform changed in concert with the angular motion. This was accomplished by first converting the previous threshold- and background-subtracted frame into a binary image (pixel values 1 or 0). A binary close operation (dilation followed by erosion) was then performed on the image, using ImageJ to fill in the residual gaps created by the background grid and creates a continuous shape outlining the incident spot area. Once this was accomplished, particle analysis, standard to ImageJ, was performed on the frame, which undertakes ellipsoid fitting to each of the closed shapes within the frame. The analysis also returned several metrics for each particle shape, including circularity, roundness, aspect ratio, and ellipse rotation angle (described further in Appendix A), the values of which were time averaged for periods of before, during and after laser illumination to capture the illumination response and any burn-in effects. Figure 11d–f show this processing graphically for a single frame. Additional information on this particle analysis can be found in the ImageJ online documentation [48]. For the data in these experiments, only the primary central shape was tracked and any small particles on the periphery of the spot were ignored.

## 5. Results and Discussion

Experimental characterization of the photothermal response was performed on several structures, with results from two baseline (devices A and B) and two single-arm Cr-coated (devices C and D) structures reported in this article. These devices differed in their initial plate bifurcation, with A and C being cylindrically curved in the vertical direction and B and D being cylindrically curved in the horizontal direction. This section presents the full datasets for devices A and C corresponding to the test flows shown in Figure 9 and the images in Figure 2. The test flows and corresponding complete datasets for devices B and D are provided in the Supplementary Materials (Figures S1–S3). Videos of the laser spot data on screen and of the devices themselves are also provided in the Supplementary Materials (Videos S1–S3) for a select example test point.

**Figure 12:**
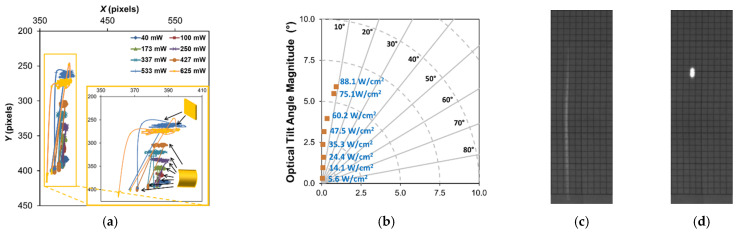

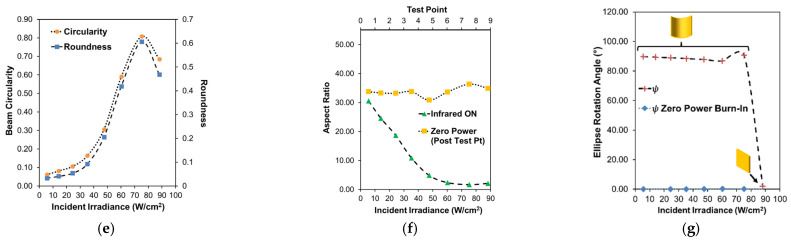
Results for device A baseline structure. (**a**) Complete center of mass positioning data with qualitative plate curvature based upon observed spot shape shown inset. (**b**) Polar plot of average spot position at each irradiance level relative to initial position at start of test point (burn-in removed). Single, cropped, perspective-rectified frame (**c**) before illumination and (d) during 75.1 W/cm^2^ illumination. After ellipse shape parameters: (**e**) beam circularity and roundness during illumination; (**f**) aspect ratio during (green triangles—bottom axis) and after illumination (i.e., zero irradiance value; yellow squares—top axis). The test point number indicates the exposure count (1 = first exposure, 2 = second exposure, etc.) per the test flows shown previously in Figure 9a. (**g**) Ellipse rotation angle indicating plate curvature direction and corresponding burn-in, which was near zero.

The angular (tilt) response for the two baseline structures was similar in magnitude but differed in direction. Figure 12a–g summarize the results for device A. Figure 12a shows the calculated laser spot position on the screen for every frame for each test run. The results show clear tilt motion, preferentially in one direction (toward the top of the screen) as infrared irradiance is increased, with some cross-axis coupling (lateral motion) at higher laser powers shown in Figure 12b. Since the support arms were all uncoated, the differing preference in tilt direction can be attributed to a combination of the asymmetric deformation of the central plate and interconnect supports, as well as imperfect positioning of the beam. For device A, a maximum mechanical tilt, averaged across a single exposure, of ~3° (6° optical) was shown for 75 W/cm^2^, with position relaxing slightly at 88.1 W/cm^2^. This decreased motion for the higher exposure irradiance was possibly due to a reduction in Au film stress from thermal annealing. device B (shown in Figure S2) yielded a similar response in a lateral direction with a maximum mechanical tilt of ~3.65° (7.3° optical) for 74.2 W/cm^2^ , which was the maximum power response successfully captured (a higher exposure of 87.8 W/cm^2^ was performed but the response exceeded the limits of the screen). While there was clearly some significant tilt burn-in effects (i.e., plastic deformation resulting from the infrared exposures, evident in Figure 12a), pre- and post-imaging comparison (not shown) suggested no damage to the structural profile post-testing. Both the center height position and the radius of curvature of the plate remained unchanged and the differences in both central plate tip and tilt angles were <0.1°. Thermomechanical burn-in has been studied before, specifically for Au on polysilicon plate structures, by Gall et al. [49], and thus it is not surprising to see some burn-in response in these large-deformation structures that are dependent in part on the stress in the Au layer. Lastly, as each exposure increased in power, there was clearly some drift observed in the position on the beam during the exposure. This drift suggests a slow temperature rise, most likely inherent to the local thermal boundary conditions changing, the most significant being heating of the substrate.

**Figure 13:**
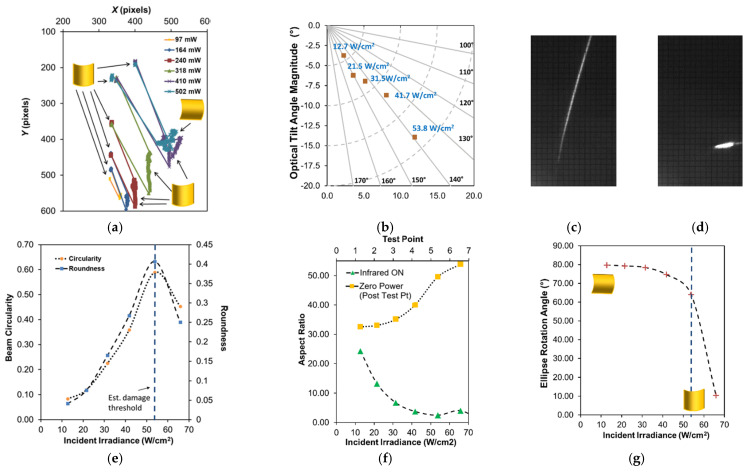
Results for device C single-arm Cr-coated structure. (**a**) Complete center of mass positioning data with qualitative plate curvature based upon observed spot shape shown inset. (**b**) Polar plot of average spot position at each irradiance level (prior to estimated damage onset) relative to initial position at start of test point (burn-in removed). Single, cropped, perspective-rectified frame (**c**) before illumination and (**d**) during 53.8 W/cm^2^ illumination. After ellipse shape parameters: (**e**) beam circularity and roundness during illumination; (**f**) aspect ratio during (green triangles—bottom axis) and after illumination (i.e., zero irradiance value; yellow squares—top axis). The test point number indicates the exposure count (1 = first exposure, 2 = second exposure, etc.) per the test flows shown previously in Figure 9b. (**g**) Ellipse rotation angle indicating plate curvature direction and corresponding burn-in, which was near zero. The dashed blue line is shown to indicate estimated damage threshold.

**Figure 14:**
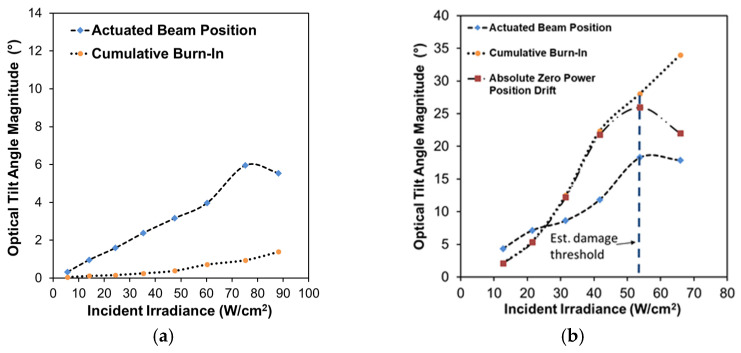
Relative actuated beam position (burn-in removed) and cumulative burn-in from initial tilt at start of testing: (**a**) device A (baseline), (**b**) device C (single-arm Cr-coated) with the absolute position burn-in drift added as an indicator of damage onset (marked by the dashed blue line).

The results for single-arm Cr-coated structures are shown in Figure 13 (device C) and Figure S3 (device D). Overall, these structures exhibited a larger magnitude tilt response, in comparison to the baseline structure, towards the direction of the coated arm, as shown in Figure 14 and Figure S4, which was coincident with the designed thermal absorption asymmetry. They also exhibited a much larger degree of burn-in motion (see Figure 13a, Figure 14, and Figure S4b) and drift motion during exposure, likely due to both longer exposure times (40–50% longer in comparison to baseline) and the aforementioned flaws in the Cr coating, namely localized delamination, allowing for increased localized heating and additional plastic deformation. Higher power exposures did eventually lead to twisting and warping of the coated arm, verified by imaging post-experiment. While this damage onset was not immediately obvious from the direct observation of the device, examination of the post-processed data showed the onset of aberrant cross-axis drift motion and burn-in of the spot.

One way to quantify this damage effect is by comparing the deviation of the cumulative burn-in position to the absolute burn-in position in optical tilt angle magnitude, as shown in Figure 14b and Figure S4b. The cumulative burn-in is the cumulative summation of burn-in observed for all exposures before and including the given exposure test point. The absolute burn-in is the absolute spot distance relative to the initial spot position observed at the beginning of the experiment before any exposures were performed. Deviations between these two values indicate there is a change in the direction of the burn-in motion, which suggests twisting deformation damage of the structure is occurring. The maximum mechanical tilt observed for device C, prior to (or perhaps at) the onset, was ~9.15° (18.3° optical) for 53.8 W/cm^2^. Device D (shown in Figure S3) was very similar in magnitude, although with less cross-coupled motion (a ~12° vs. ~40° off-perpendicular screen in-plane trajectory), yielding a ~8.65° (17.3° optical) maximum tilt angle at 53.7 W/cm^2^. While the steady-state simulation results in Section 3 agree qualitatively, in terms of the general direction of the response, these measured tilt magnitudes are much larger in comparison, suggesting that the effective parameters used to capture the initial deformation are not well-suited for the complete photothermal response.

The corresponding observed laser spot shape responses for both baseline structures (Figure 12e–g and Figure S3e–g) and the single-arm Cr-coated structures (Figure 13e–g and Figure S3f–h) was qualitatively consistent with expectations associated with heating of layered plates. The expected thermomechanical response for a centrally anchored metal-coated plate, as described by Dunn et al. [36], would be a deformation transition from a convex to concave deformation as the plate temperature is increased. In all structures, the beam shape consistently became more circular as the irradiance increased, per the circularity and roundness plots (Figure 12e, Figure 13e, Figures S2e and S3h), which implies flattening of the central plate due to a relaxation of the Au thermal stress. This trend was consistent for the baseline structures, although there was some small deviation in the circularity, roundness and rotation angles produced from the ellipsoid fitting at elevated irradiances (Figure 12e,g and Figure S2h), possibly from some asymmetric plate anchor loading with the surrounding interconnect and support arms impeding the plate deformation. However, for the Cr-coated structures, there was an abrupt change in the orientation of the spot ellipsoid (see rotation angle plots in Figure 13c,d and Figure S3d) that occurred in the structures before the estimated damage thresholds. This change in rotation angle corresponded to a change in the direction of the center plate bifurcation, likely due to exaggerated asymmetry of the edge anchor loading of the plate to the support arms triggering the bifurcation to “snap” in the opposite direction. This behavior seemed to be independent of the initial direction as devices C and D were each initially oriented opposite each other and it occurred in both. The plots in Figure 12f, Figure 13f, Figures S2f and S3gt both the aspect ratio of the laser spot during illumination of a given exposure (green triangles) and the aspect ratio of the laser spots after illumination (yellow squares). The intent is to portray any permanent distortion of the laser spot shape, which would indicate plate deformation (i.e., burn-in) of the central plate. For both the baseline structures, there was no significant burn-in effect associated with the beam shape (plate curvature) per the aspect ratio plots (Figure 12f and Figure S2f). However for the single-arm Cr-coated structures, there was some drift in the zero power aspect ratio (Figure 13f and Figure S3h). Since beam shape burn-in was completely absent in the baseline structures, the burn-in observed was most likely a consequence of asymmetric burn-in deformation occurring in the support arms of the single-arm Cr-coated structure rather than the plate itself.

While this experiment could not measure piston motion directly, significant piston motion was clearly evident from the device video recordings for all structures tested (see Video S1). The response was consistently observed when the power was fixed and the laser was moved across the elements of the uncoated array. It is also worth noting that the intended tilt behavior of the single-arm coated structures could be altered through deliberate off-center alignment of the incident beam, although we do not present these results.

## 6. Conclusions

The results presented in this article clearly show that large-deformation thin-film structures have the ability to be actuated for significant tilt angles remotely through the use of infrared laser illumination. Deliberate tilt actuation was demonstrated for symmetric structures using inherent imperfections in the off-center beam position, although the exact positioning of the beam offset was not measureable with the test setup employed. Alternatively, we demonstrated that a more responsive tilt actuation can also be generated through deliberate application of asymmetric infrared absorption across the structure in the presence of a fixed, centrally positioned laser beam. The latter exhibited significantly more (5–10× in comparison to the uncoated baseline) burn-in (plastic non-recoverable) deformation, albeit with longer exposure times (40–50% longer), than the baseline comparison cases. This burn-in effect can be largely attributed to accentuated localized heating from film defects (namely delamination) of the metal infrared absorber layer causing a greater degree of both film annealing and thermomechanical deformation.

A more exhaustive investigation involving device response cycling and full hysteresis testing remains to be accomplished for these structures, but the current data and responses observed suggest that at lower powers the response may be repeatable for a fixed exposure time. Burn-in is something that is common to encounter in both microsystems and microelectronics and is often accommodated through in-situ or externally applied pre-heat treatment (annealing). For applications that may seek to reduce this burn-in effect, one clear approach would be employing a thinner Cr coating or perhaps an alternate material that will have less coupling to the structural stiffness without compromising absorption or the desired photothermal response. In additional, tensile thin films other than metal may be employed to reduce or even eliminate the known burn effects associated with annealing of ductile metal films, such as the Au employed here. Targeted design of these types of structures for steady-state photothermal response operation requires careful consideration of the films used and the geometry employed to manage photothermal sensitivity, total range of motion and nonlinear effects, such as burn-in of the structures. The photothermal response of these structures was demonstrated at power levels that would be considered high for many applications, but the potential to tailor for a lower power response remains possible through targeting structures with lower effective stiffness with coatings that promote higher infrared absorption.

Design of structures similar to these for beam steering/shaping applications demands the ability to generate accurate predictions of their behavior. Ultimately, the simulations developed in this study are effective for qualitative comparison and concept demonstration, but are insufficient for accurate prediction of deformation profiles, coupled photothermal response or accommodation of nonlinear effects such as burn-in. Capturing these effects appears to be possible with existing multiphysics tools but remains an area to be fully explored.

## Figures and Tables

**Table 1:** Properties of thin-film materials used for simulations.

Material Properties	Materials			
	Au	Polysilicon	Si_3_N_4_	Cr ^†^
Young’s modulus (GPa) ^◊^	70	160	135 (20) ^†^	279
Poisson’s ratio	0.44	0.22	0.23	0.21
Density (kg/m^3^)	19,300	2320	3100	7150
Applied film stress (MPa) ^◊^	65	−600	−1000	100
Coefficient of thermal expansion (10^−6^/K)	14.2	2.6	2.3	4.9
Thermal conductivity (W/(m·K))	317	34	20	93.7
Heat capacity at constant pressure (J/(kg·K))	129	678	700	448
Emissivity (absorption) at 1532 nm	0.03	0.058 ^‡^	N/A	0.30–0.40

^†^ For the single-arm Cr-coated model only; ^‡^ applied to POLY2/Si_3_N_4_ film stack; ^◊^ “effective” values used.

**Table 2:** Summary of devices tested.

Device Name	Device Type	Central Plate Curvature Orientation	Exposure Times
A	Baseline	Vertical	7–10 s
B	Baseline	Horizontal	7–10 s
C	Single-arm Cr-coated	Vertical	14–15 s
D	Single-arm Cr-coated	Horizontal	14–15 s

**Figure A1:**
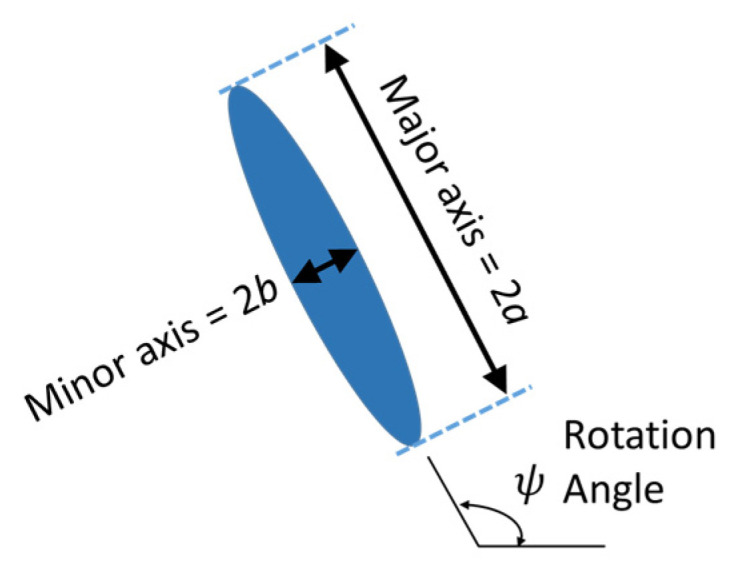
Fundamental ellipse parameters.

## Supplementary Materials

The following are available online at https://www.mdpi.com/article/10.3390/mi12040428/s1, Figure S1: Additional test flows executed with exposure times; Figure S2: Results for baseline structure device B; Figure S3: Results for baseline structure device D; Figure S4: Relative actuated beam position and cumulative burn-in results for devices B and D. Video S1: Device D post-processed grayscale dataset at 53.7 W/cm^2^ “DeviceD_TP4_53W_cm2_Final.avi”; Video S2: Device D ellipsoid-fitted dataset at 53.7 W/cm^2^ “DeviceD_TP4_53W_cm2_Particle.avi”; Video S3: Device D microscopy video at 53.7 W/cm^2^ “DeviceD_TP4_53W_cm2_DirectVideo.wmv”.

## Appendix A—Ellipse Shape Parameters

For a given ellipse with rotation angle Ψ, as shown in Figure A1, the aspect ratio, circularity and roundness are defined as:

(A1) $$\mathrm{Aspect}\;\mathrm{ratio}=\frac{a}{b}$$

(A2) $$\mathrm{Circularity}=\frac{4\pi \left[Area\right]}{{\left[Perimeter\right]}^{2}}$$

(A3) $$\mathrm{Roundness}=\frac{b}{a}$$

Each of these parameters was determined for the binary screen images using routines internal to ImageJ. Per the online documentation [48], a best-fitting ellipse algorithm (EllipseFitter.java) can be used to determine *a* and *b*, and aspect ratio and roundness are thus directly calculated. The area and perimeter used for circularity are not specific to the ellipse shape and are instead determined through more generic binary image calculations. The area is simply the number of 8-neighbor connected pixels within the shape and the perimeter is determined through weighted length values associated with edge pixels and corner pixels (the getTracedPerimeter method in the PolygonRoI.class). Ellipse area can also be calculated directly as:

(A4) Area=πab

and the best-fitting ellipse algorithm uses this calculation to best match the calculated area of the shape to that found through pixel summation.
