# Supplementary material for: Photothermal Optical Beam Steering Using Large Deformation Multi-Layer Thin Film Structures
Source: Micromachines (Basel). 2021 Apr 14;12(4):428. doi: 10.3390/mi12040428 (PMC8070700; doi:10.3390/mi12040428)
Supplement: Supplementary file 1 [file micromachines-12-00428-s001.zip › supplementary/micromachines-1133042-supplementary.pdf]

Supplementary Materials:

There are four supplementary figures (Figures S1-S4) and three supplementary videos (Videos S1-S3) provided. The Figures provide all the supporting test flow and results for Devices C and D as indicated in the narrative. The videos all pertain to the Device D, 53 W/cm^2 (409 mW laser power) 13.7 ms 1532 nm infrared laser test exposure. Video S1 shows the perspective rectified greyscale video of the visible laser spot on screen, Video S2 the ellipsoid fitted video, and Video S3 is the recorded optical microscopy video, which shows the actuation and piston observed for the structure. Video S1 shows the clear change off the beam from an elongated horizontal profile to a slightly oblong vertical ellipse upon infrared illumination. There is some slow beam drift occurring during the exposure and the beam position after the exposure is clearly offset from the original initial position showing evidence of burn-in. In Video S2 the initial unilluminated fitted ellipsoid is split in two for many frames before exposure vs. after exposure. This splitting of the primary spot into two ellipsoids was very rare and was not of primary concern as it did not occur for any data during infrared illumination. However, it is one potential flaw that can occur with using this approach in capturing surface curvature changes.

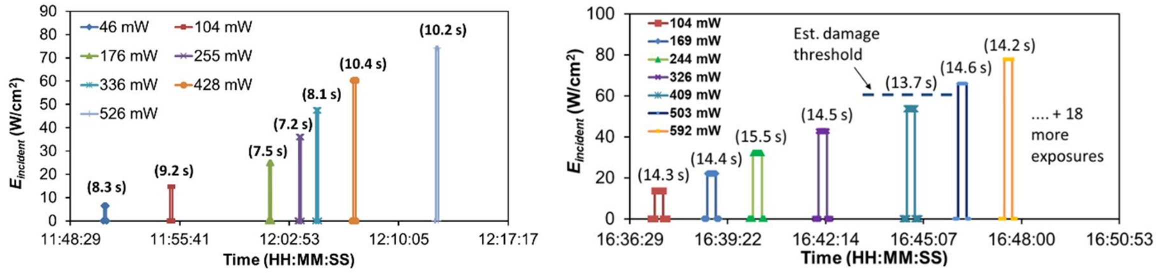

(a) **(b)**

Figure S1. Additional test flows executed with exposure times (a) baseline structure (no Cr coating – Device B) with ~7-10 sec exposure times (b) single arm Cr coated structure (Device D) with ~14-15 sec exposure times

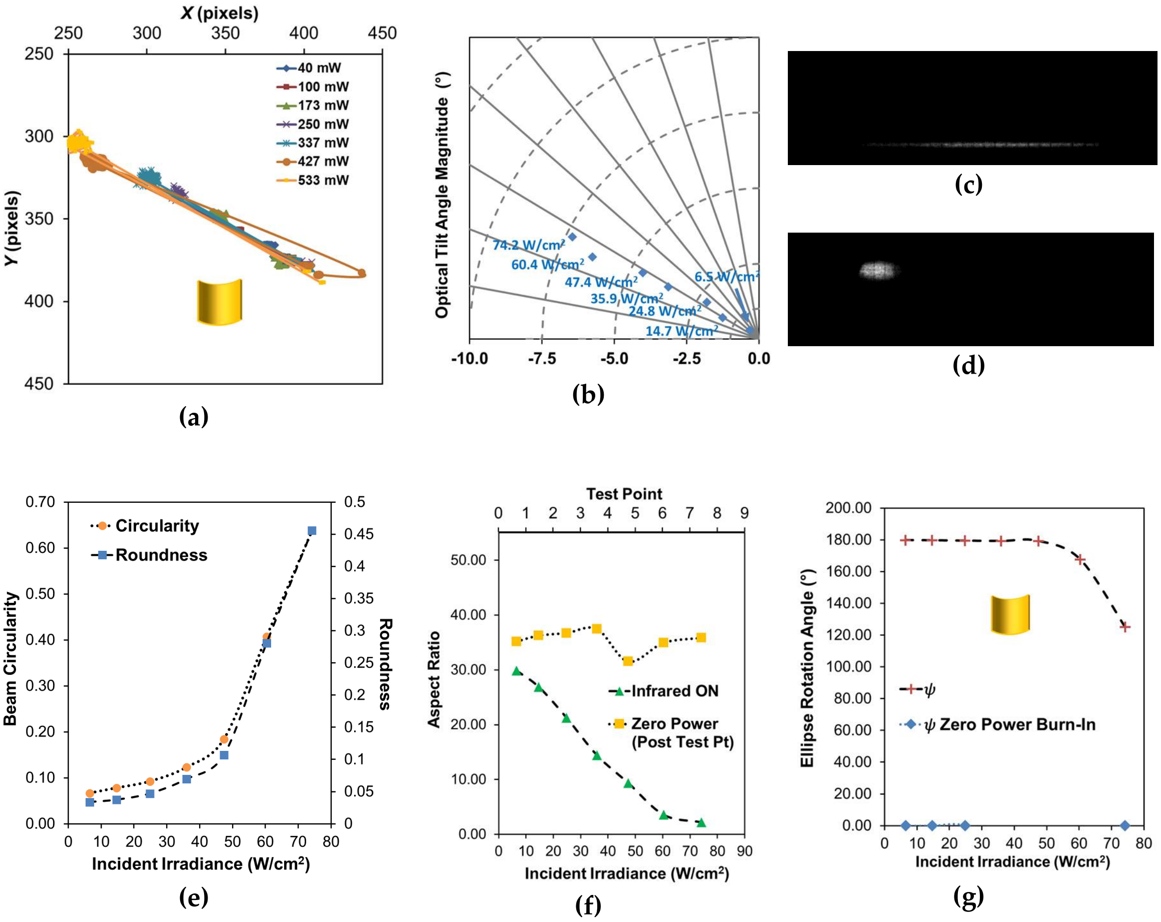

Figure S2. Results for baseline structure Device B (a) Complete Center of Mass positioning data with qualitative plate curvature based upon observed spot shape shown inset (b) Polar plot of average spot position at each irradiance level relative to initial position at start of test point (burn-in removed). Single cropped perspective rectified frame (c) before illumination (d) during 74.2 W/cm^2 illumination. After ellipse shape parameters (e) beam circularity and roundness during illumination (f) aspect ratio during and after illumination (g) ellipse rotation angle indicating plate curvature direction and corresponding burn-in, which was near zero.

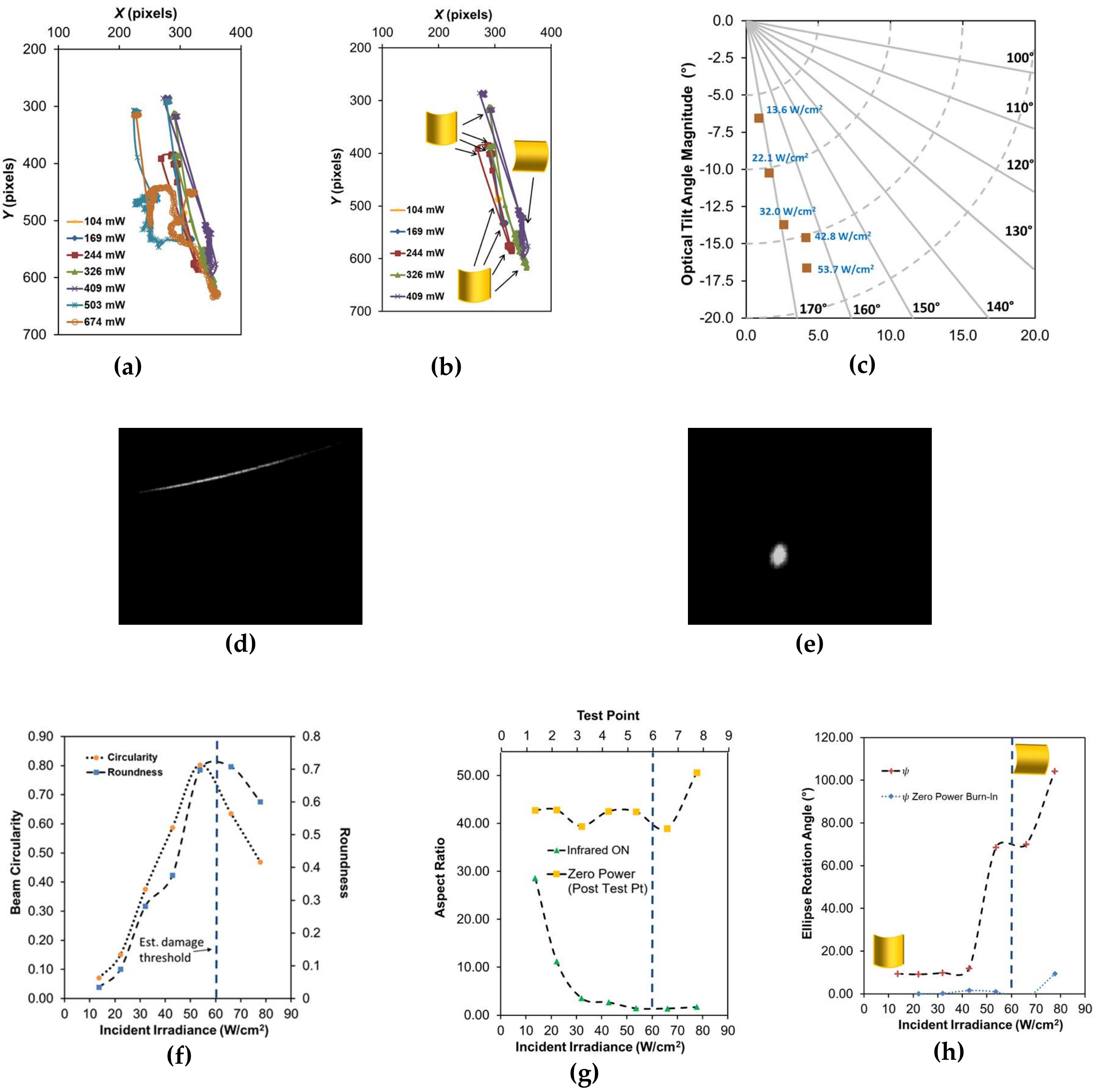

Figure S3. Results for baseline structure Device D (a) Complete Center of Mass positioning data with qualitative plate curvature based upon observed spot shape shown inset (c) Polar plot of average spot position at each irradiance level relative to initial position at start of test point (burn-in removed). Single cropped perspective rectified frame (d) before illumination (e) during 53.7 W/cm^2 illumination. After ellipse shape parameters (f) beam circularity and roundness during illumination (g) aspect ratio during and after illumination (h) ellipse rotation angle indicating plate curvature direction and corresponding burn-in, which was near zero.

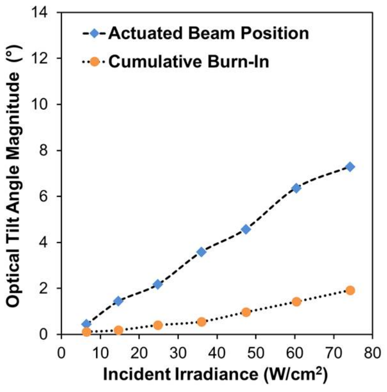

(a)

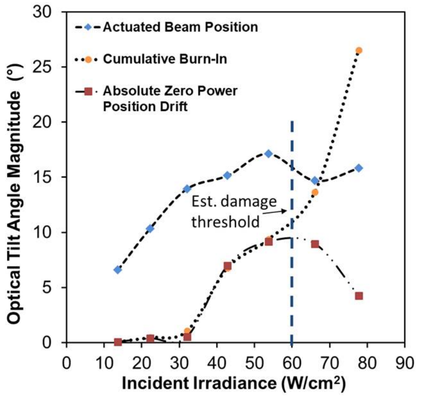

(b)

Figure S4. Relative Actuated Beam Position (Burn-In removed) and Cumulative Burn-In from initial tilt at start of testing (a) Device C (baseline) (b) Device D (single arm Cr coated) with the absolute position burn-in drift added as an indicator of damage onset. The dashed blue line indicates estimated damage threshold.
